# Minimally Invasive Multiport Surgery of the Lateral Skull Base

**DOI:** 10.1155/2014/379295

**Published:** 2014-07-02

**Authors:** Igor Stenin, Stefan Hansen, Meike Becker, Georgios Sakas, Dieter Fellner, Thomas Klenzner, Jörg Schipper

**Affiliations:** ^1^Department of Otorhinolaryngology, University Hospital Düsseldorf, 40225 Düsseldorf, Germany; ^2^Interactive Graphics Systems Group, Technical University Darmstadt, 64283 Darmstadt, Germany

## Abstract

*Objective*. Minimally invasive procedures minimize iatrogenic tissue damage and lead to a lower complication rate and high patient satisfaction. To date only experimental minimally invasive single-port approaches to the lateral skull base have been attempted. The aim of this study was to verify the feasibility of a minimally invasive multiport approach for advanced manipulation capability and visual control and develop a software tool for preoperative planning. *Methods*. Anatomical 3D models were extracted from twenty regular temporal bone CT scans. Collision-free trajectories, targeting the internal auditory canal, round window, and petrous apex, were simulated with a specially designed planning software tool. A set of three collision-free trajectories was selected by skull base surgeons concerning the maximization of the distance to critical structures and the angles between the trajectories. *Results*. A set of three collision-free trajectories could be successfully simulated to the three targets in each temporal bone model without violating critical anatomical structures. *Conclusion*. A minimally invasive multiport approach to the lateral skull base is feasible. The developed software is the first step for preoperative planning. Further studies will focus on cadaveric and clinical translation.

## 1. Introduction

Today, minimally invasive procedures (MIPs) are well established in various surgical fields. In MIPs, small incisions and miniaturized instruments are used to minimize iatrogenic tissue damage. MIPs lead to a shorter length of hospital stay, less postoperative pain, earlier postoperative recovery, and a lower complication rate compared with open access surgery [1–3]. While MIP approaches to the frontal skull base are increasingly used, surgery of the lateral skull base still requires a wide exposure of the anatomical landmarks and the intraoperative, direct identification of the critical anatomical structures by the surgeon. As an example, the common surgical technique of cochlear implantation is mainly based on mastoidectomy and posterior tympanotomy.

Minimally invasive, single-port approaches to the lateral skull base have been attempted by several authors using neuronavigated drilling [4–9]. Bell et al. developed an image-guided robot and performed the drilling of one access tunnel for the insertion of an electrode in a temporal bone specimen [6]. Labadie et al. demonstrated percutaneous single-port access to the cochlea in vitro and in vivo using preoperative computed tomography, bone-implanted fiducial markers, and a customized microstereotactic frame [8, 10]. This concept was further extended by Wanna et al., who created percutaneous single-port access to the petrous apex in a cadaveric temporal bone specimen using the same setup [9].

**Figure 1:**
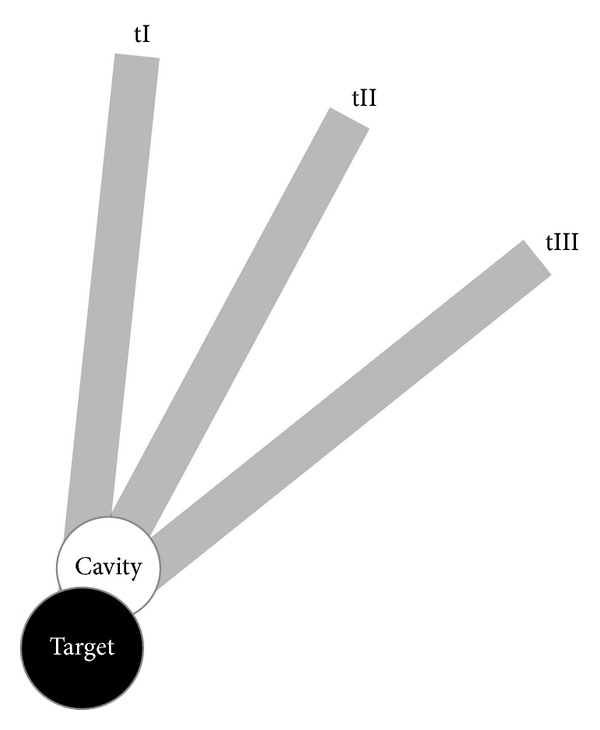
Multiport approach: intersection of the trajectories (tI–III) at the target and the cavity.

In contrast, we envision a minimally invasive* multiport* setup that provides access to the lateral skull base similar to that used in laparoscopic surgery, with one port for visualization via an endoscope and two ports for instruments. The use of three ports enables advanced manipulation, direct visual feedback, and use for more indications compared with a single-port approach. Furthermore, the intersection of the three trajectories at the target forms a cavity, which creates additional space for manipulation around the site of interest (Figure 1). Possible indications are tumor removal, biopsy, drug delivery, brachytherapy, or cochlear implantation via two instruments and under direct endoscopic visual control. Liu et al. showed the possibility of da Vinci Si-assisted cochlear implant surgery with augmented reality in cadaveric feasibility study [11]. The planning and future realization of multiple minimally invasive ports could be used to implement a similar master slave system. Prior to drilling, the location and direction of the ports must be planned and simulated with preoperative planning software utilizing adequate radiological data. The outcome of the planning software should be (1) clarification of the general feasibility of such an approach preoperatively, (2) prediction of the possible diameters and angles of all three canals, and (3) calculation of trajectories that do not violate critical neurovascular structures.

The aim of this study was to develop and evaluate a software tool for a patient-specific determination of three collision-free trajectories (CFTs) toward various targets within the temporal bone. We developed a planning tool for multiport surgery of the lateral skull base, which facilitates the planning of trajectories inside patient-individual three-dimensional (3D) temporal bone models derived from preoperative computed tomography (CT) scans. Using the planning tool, we analyzed the feasibility of three trajectories, the position of the trajectories, and the maximum drill diameters in twenty different native CT scans of the temporal bone. The internal auditory canal (IAC), round window (RW), and petrous apex (PA) were chosen as representative target points. Possible applications are biopsy and resection of benign and malignant lesions (e.g., metastases, vestibular schwannoma, and paraganglioma), drug delivery (e.g., biologicals, stem cells), drainage of cholesterol granuloma, or cochlear implant insertion. A set of three CFTs was simulated for each target without violating critical structures.

## 2. Materials and Methods

### 2.1. Preprocessing

**Figure 2:**
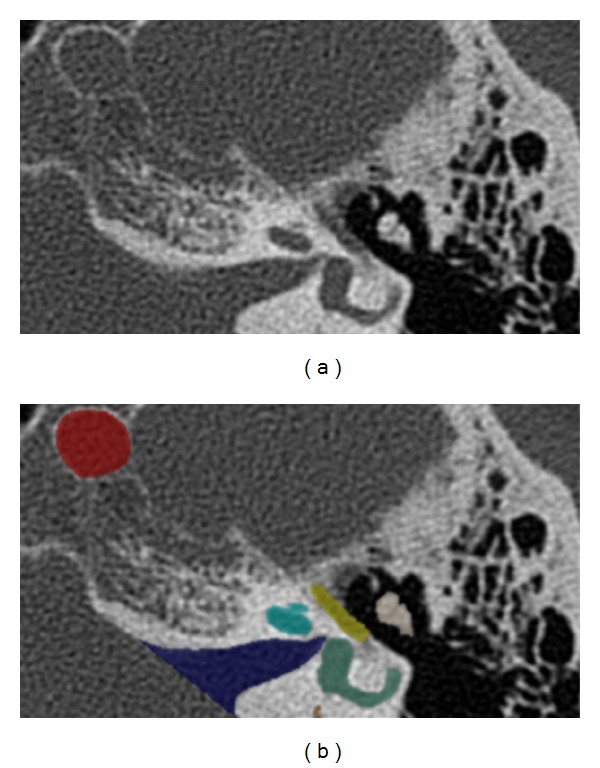
Manual segmentation of the anatomical structures: the left (a) image shows the axial plane of a temporal bone CT scan. The internal carotid artery (red), internal auditory canal (blue), cochlea (teal), labyrinth (green), facial nerve (yellow), and ossicles (beige) were manually segmented in the right (b) image.

To plan and simulate a minimally invasive multiport procedure in the temporal bone, individual anatomical 3D models were extracted from actual CT image data. Twenty regular CT scans obtained using a standard clinical scanning protocol for temporal bone (Siemens Somatom, Siemens, Eschborn, Germany) average resolution 0.19 × 0.19 × 0.39 mm^3^ were used. The following anatomical structures were defined as essential: the internal carotid artery and jugular vein bulb, facial nerve and chorda tympani, cochlea and labyrinth, internal and external auditory canal, ossicles, brain, and cranial bone. All structures except for the cranial bone and brain were manually segmented with the ITK-Snap freeware software [12] within each slice of the CT scans (Figure 2). The cranial bone was automatically extracted using simple thresholding. For segmentation of the brain, a safety margin was extracted semiautomatically using a pixel-based approach. It is specifically designed for a certain volume of interest and uses a ray casting approach. The segmentations were verified and manually corrected via visual control of all processed slices by an experienced skull-base surgeon. The chorda tympani could not be identified in one dataset. After segmentation, a triangle mesh was extracted using the Marching Cubes algorithm [13]. The triangle meshes of the anatomical structures were loaded into the planning tool.

### 2.2. Planning Tool and Selection of Drill Path Combinations

**Figure 3:**
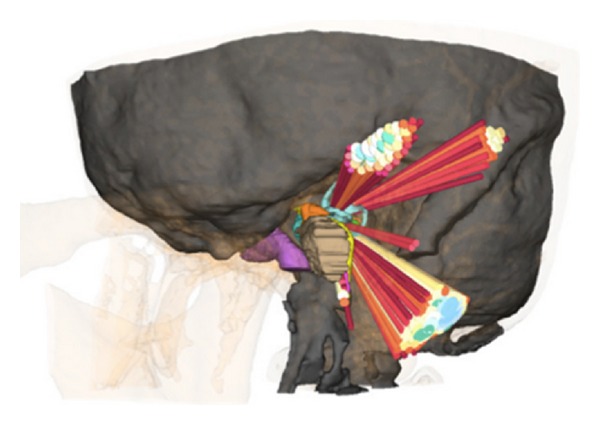
Color-coded CFTs to the internal auditory canal; reddish (hot) colors indicate a small and bluish (cold) colors indicate a larger distance to the risk organs.

Our planning tool software uses the public domain physical simulation C++ library “Simulation Open Framework Architecture” (SOFA) [14] running on a standard personal computer with a Windows operating system. For CFT planning, a custom graphical user interface (GUI) was added to SOFA. The protocol was as follows: first, a region for candidate entry points of the trajectories is defined on the surface of the 3D triangle mesh skull model. By clicking on the surface of the model the center point of the area of candidate entry points is marked. Then all triangle center points of the skull's triangle mesh within a user defined distance to the chosen point are added to the set of candidate entry points. The target of the trajectories can be defined by clicking on a point in the 3D model or inside the axial CT image. Furthermore, the GUI allows for definition of a drill path radius, a minimum safety distance to the critical structures, and the inaccuracy of the drill. After determining all variables, the software computes and displays all CFTs from the entry points at the skull to the target point, fulfilling the above constraints. The CFTs are color-coded based on their distance to the closest critical structure as follows: the reddish (hot) color indicates a small distance, which is associated with higher risk drillings, whereas the bluish (cold) color indicates a larger distance to the risk organs, that is, safer paths (Figure 3). The planning software allows the definition of an individual safety margin (minimum safety distance to the critical structures and the inaccuracy of the drill). The required value of the safety margin highly depends on the actual clinical setup, for example, the quality of the CT scan, the fiducial markers, drilling process, and so forth. Therefore we decided not to set a fixed safety margin value in this general feasibility study.

**Figure 4:**
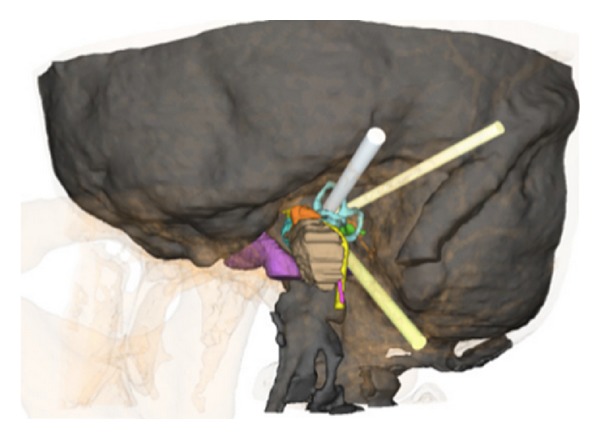
A set of three CFTs selected by the evaluator.

A set of three CFTs can then be selected by clicking on the displayed trajectories. The GUI displays the angles between the paths, the length of the individual paths in millimeters (mm), and the distance to the closest critical anatomical structure in mm (Figure 4). Two criteria were considered in the selection of the optimal CFTs: the maximization of (1) the distance from each drill path to critical structures and (2) the distance between the entry points, that is, the angles between the CFTs (see the next section).

The planning of a set of three CFTs was performed according to the above-described protocol in each of the twenty patients, targeting the RW, IAC, and PA. The drill path diameter was set to 1 mm in each case. The sum of the defined diameter and the safety distance in mm represents the largest possible diameter of a CFT. Thus, the resulting set of CFT does not necessarily have the same diameters. The diameters can be varied as needed in a future intervention, for example, one small diameter for the endoscope and two larger diameters for instruments. In each case, the target structure itself was labelled as noncritical and was therefore not considered for collision detection. We envision the implantation of a cochlear implant as the main indication for targeting the RW. Therefore, the ossicles were labeled as noncritical, as residual hearing is not relevant in this case.

### 2.3. Analysis of Virtual Drilling Canals and Statistics

The software SPSS (Statistical Package for the Social Sciences) 11 (IBM, USA) was used to perform the statistical analysis and generate the graphs. Boxplots were employed to account for the individual anatomical variations and the consequential differences in the location and diameter of the drill paths. In the following section, we show the median diameter of the drill paths, along with the first and third quartiles, the minimum and maximum values, and the outliers. The data are displayed separately for each target area and subdivided by anatomical region. Ultimately, the cumulative values of each anatomical region are presented. Three angles were calculated between corresponding pairs of the 3 CFTs, and the degrees of these three angles were summed. We termed the resulting value the cumulative angle (CA) and used this value as an indication benchmark. Specifically, a high CA value indicates the preferable configurations because the drill paths are further spread apart, and, therefore, the intersection of the paths occurs at a closer distance to the target.

## 3. Results

### 3.1. Software and Protocol

**Figure 5:**
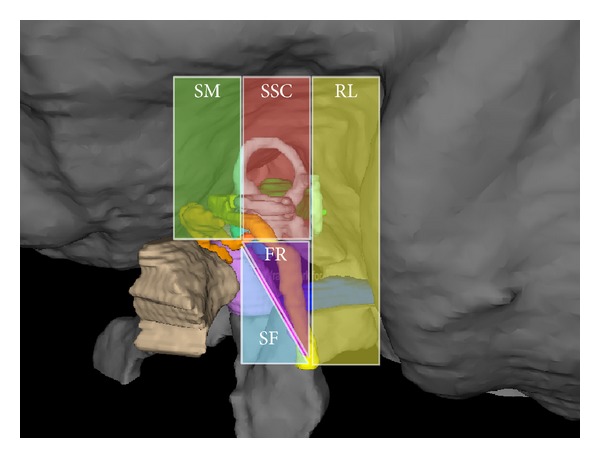
Five anatomic regions for passing CFTs: suprameatal (SM), superior semicircular canal (SSC), retrolabyrinthine (RL), chorda-facial recess (FR), and subfacial (SF).

Our software and protocol proved to be stable. The software was optimized by clinician feedback during the study in terms of workflow and user comfort. For example, the color labeling was added and the user interface was developed in close cooperation between clinician and computer scientists. CFTs to the three target structures were calculated for all 20 patients. Of all the generated CFTs, a set of three was chosen by employing the color-coding scheme and considering the displayed angles between trajectories. To enable a more intuitive evaluation of the results, we defined the following five anatomical regions for better visualization and presentation: suprameatal (SM), superior semicircular canal (SSC), retrolabyrinthine (RL), chorda-facial recess (FR), and subfacial (SF) (Figure 5).

### 3.2. Collision-Free Paths and Diameters

**Figure 6:**
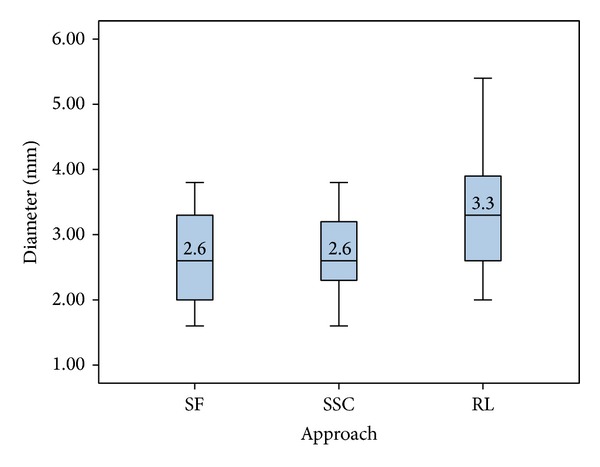
Diameters and anatomical regions of the CFTs for the IAC target point (subfacial (SF), superior semicircular canal (SSC), and retrolabyrinthine (RL)).

In each of the 20 cases, the evaluators determined three CFTs to the IAC, representing three alternative and valid configurations for each patient. Of all 60 defined paths to the IAC, 53% were inside the retrolabyrinthine area, 40% passed through the SSC, and 23% passed subfacially. The region with the largest possible median drilling diameters was found to be the retrolabyrinthine (3.3 mm), followed by the SSC (2.6 mm) and the SF area (2.6 mm). Overall, the collision-free path with the largest diameter (5.4 mm) was located in the retrolabyrinthine, and the CFT with the smallest diameter (1.6 mm) was located subfacially and in the SSC (Figure 6).

**Figure 7:**
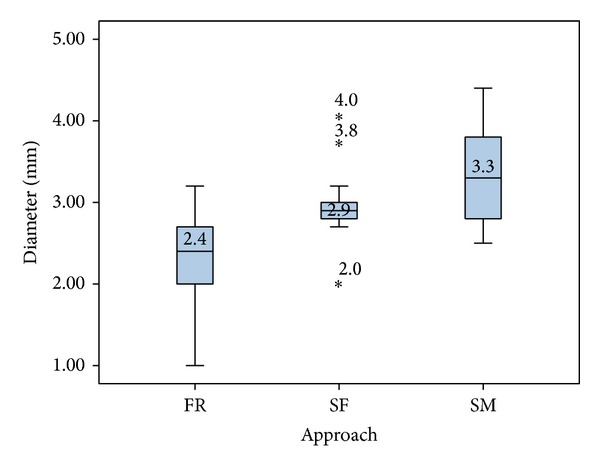
Diameters and anatomical regions of the CFTs for the RW target point (facial recess (FR), subfacial (SF), and suprameatal (SM)).

Three collision-free drill paths to the RW were also found in all 20 cases. The 60 possible paths were localized in the FR, SM, and SF in 33%, 43%, and 23% of all calculated CFTs, respectively. The largest median diameters were found in the suprameatal area (3.3 mm), followed by the subfacial area (2.9 mm) and the facial recess (2.4 mm). The collision-free path with the overall largest diameter passed through the SM area (4.4 mm), while the path with the smallest diameter was found in the facial recess (Figure 7).

**Figure 8:**
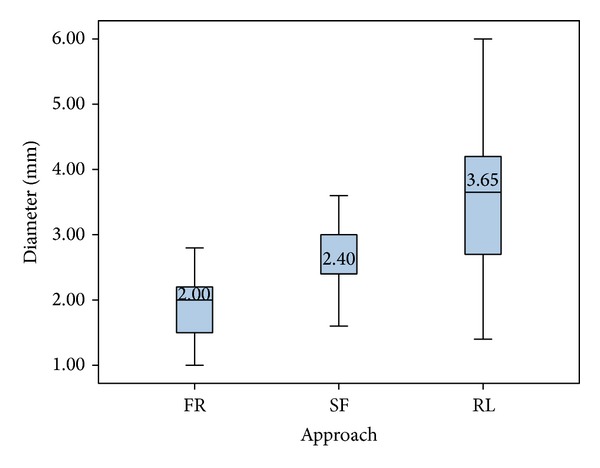
Diameters and anatomical regions of the CFTs in mm for the PA target point (facial recess (FR), subfacial (SF), and retrolabyrinthine (RL)).

Last, we simulated three CFTs to the PA in all 20 cases. The most common region to intersect with the CFTs was the retrolabyrinthine (67%), followed by the subfacial area (22%) and the facial recess (12%). The median CFT diameters for the RL, SF, and FR were 3.7, 2.4, and 2.0 mm, respectively. The collision-free path with the overall largest diameter (6.0 mm) was found in the retrolabyrinthine area and the path with the smallest diameter (1 mm) passed through the facial recess (Figure 8).

**Figure 9:**
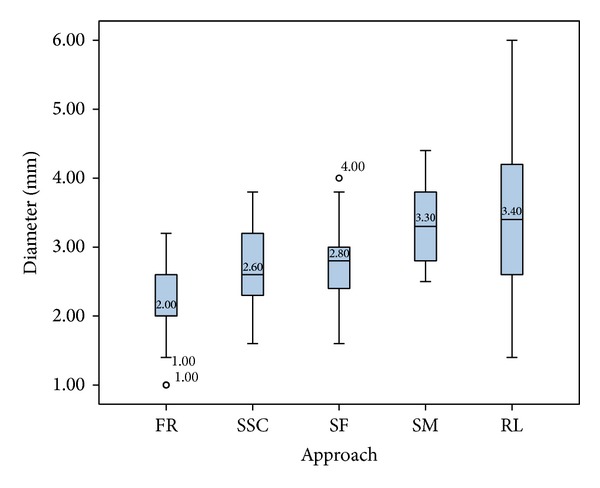
Diameters of the CFTs for all three target points by anatomical region/approach (facial recess (FR), superior semicircular canal (SSC), subfacial (SF), suprameatal (SM), and retrolabyrinthine (RL)).

To evaluate the appropriateness of each of the 5 anatomical regions as an entry-point area, we evaluated each region for all targets combined to determine the average available space for CFTs in that region. The retrolabyrinthine region showed the largest possible average diameter (3.4 mm). It is notable that this region had not only the largest maximum diameter (6.0 mm) but also the largest range, with a minimum diameter of 1.4 mm. The suprameatal, subfacial, and SSC regions allowed for mean diameters of 3.3, 2.8, and 2.6 mm, respectively. The facial recess approach provided the smallest space, with a mean CFT diameter of 2.0 mm and a minimum diameter of only 1 mm (Figure 9).

The CA was dependent on the target. For example, the largest average CA of 112° was found when targeting the RW. The IAC and PA targets displayed smaller average CAs of 98° and 40°, respectively (Table 1).

## 4. Discussion

In this study, we developed a software program for the planning and evaluation of multiple trajectories toward a designated target within the temporal bone. Based on 20 conventional CT scans of the temporal bone, it was possible to calculate and visualize for each patient a set of three alternative CFTs to the RW, internal auditory canal, and petrous apex (i.e., 9 CFTs per patient) without violating critical structures. The CFTs were chosen with the aim of maximizing both the drilling diameter and the angles between CFTs. The CFTs originated in five anatomical regions with an average measured diameter of 3.4 mm for the RL, 3.3 mm for the SM, 2.8 mm for the SF, 2.7 mm for the SSC, and 2.1 mm for the FR region. The diameters exhibited high variability, especially in the deeper regions of the temporal bone (internal auditory canal and petrous apex), because of anatomical variations, such as a high sinus bulb, low dura, or narrow chorda-facial recess.

The current planning process from a regular CT scan to the three CFTs requires approximately 2 hours. This includes manual segmentation, mesh extraction, transfer, and planning. The most time-consuming procedure is the manual segmentation. The first procedures required approximately twice as long. There was a learning curve since (i) segmentation times improved, especially when using a graphic tablet, and (ii) the surgeons gained experience with the workflow and the user interface of the software. The last step, planning of the trajectories with the SOFA planning tool, usually requires less than 15 minutes. We are currently working on semiautomatic segmentation of all anatomical structures and automatic transfer into SOFA in order to improve the duration of the process.

**Figure 10:**
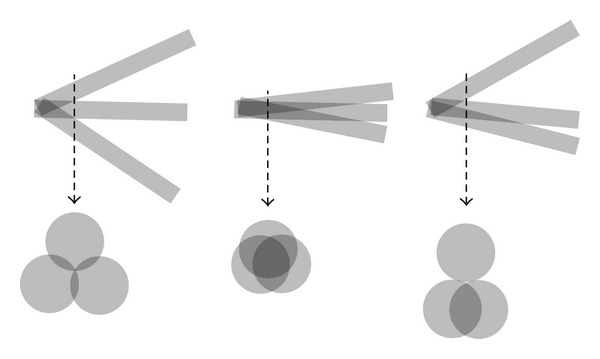
Different possibilities of intersection point of the canals.

The data demonstrate the feasibility of a multiport strategy within the temporal bone. Although several authors have demonstrated single-port approaches for MIPs in the skull base, to the best of our knowledge, a MIP multiport approach with three ports in the lateral skull base has not been previously reported. As stated above, the rationale for employing three ports is that this approach provides advanced manipulation capability and visual control. Although we were able to find three alternative, valid CFTs for each patient and target region in all 20 cases, the following aspects should be considered (Figure 10). In the multiport setup, the trajectories inevitably intersect at a variable distance before they reach the actual target. The intersection point depends on the diameter and the intertrajectory angles. A distal intersection occurring immediately at the target forms a cavity and creates additional space for manipulation around the target, which is desirable. An intersection that is too proximal may lead to fusion into two or even one path and, possibly, the loss of space for one or two instruments. The optimal relationship between the diameter and angle of the trajectories has not yet been evaluated.

The navigation, imaging, segmentation, and drilling process all have a major impact on the success of a procedure. High-resolution imaging and navigation are key factors. The mean target registration error (TRE) for commercially available otolaryngologic navigation systems with skin-affixed fiducial marker registration or bone-implanted fiducials has been reported to be at least 0.91 ± 0.28 mm [15, 16]. The use of microstereotactic frames allows for further reduction of the TRE to 0.45 ± 0.15 mm [17] or even 0.37 ± 0.18 mm [10]. Bartling et al. showed that submillimetric surgical navigation accuracy is possible by using flat panel-based volume computed tomography (TRE: 0.46 ± 0.22 mm) rather than 16-slice CT (TRE: 0.82 ± 0.35 mm) [18]. A recent study by Bell et al. presented an image-guided robot system for drilling of one minimally invasive tunnel to the round window for cochlear electrode insertion. The group reported an error of 0.15 ± 0.08 mm at the target [19].

Manual segmentation is the gold standard but is still dependent on the quality of the imaging and even more dependent on the experience of the surgeon. To the best of our knowledge, the intra- and intersurgeon variability and/or deviation from the “ground truth” have not been reviewed for skull base datasets and must be accounted for when defining the safety margin for the drilling paths. In the final setting, this operator-dependent error requires further reduction of the drill path diameter to increase the safe distance to risk structures. Currently, this safety strategy may unreasonably exclude certain patients from a MIP scenario due to the small diameter of at least one canal. A possible future solution would involve an automatic, validated segmentation protocol that may eliminate human error and that is currently under investigation by our group. Currently, the additional safety value can be manually adjusted, as appropriate, by our planning software and may be included in an individualized patient-specific therapeutic regimen.

The multiport MIP approach to the otobasis demands specially fitted, miniaturized instruments; surgery through tiny bony channels in a narrow space pushes the boundaries of “conventional” instruments. Emerging technologies, for example, piezosurgery, optical coherence tomography, or microendoscopy, may be applied for tissue ablation [20, 21]. The use of robotics may further enhance the precision of the operation; robotic systems can reduce human tremor, provide haptic feedback, and improve motor skills and articulation capabilities. The exact requirements for the instruments depend on the individual case; even with current instruments, applications such as biopsy and local injection of drugs (e.g., “targeted therapy”) may become possible due to the minimal trauma and predictable risks afforded by MIPs. Another challenge is the sufficient visualization of the region of interest in a rigid, complex region such as the otobasis. The ongoing miniaturization of the endoscope and improvement of the image quality may enable the use of the smallest drill channels for direct visualization; rigid endoscopes, commonly known as Hopkins rods, are currently available in diameters <0.9 mm and deliver better image quality than fiber optic and chip-on-tip technology [22].

**Figure 11:**
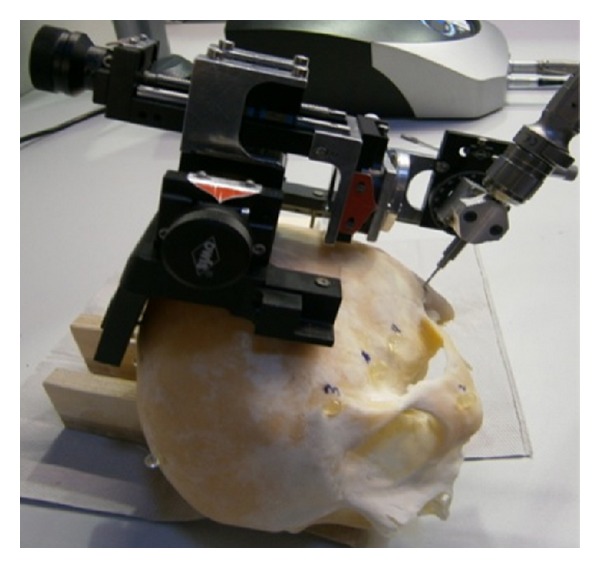
Positioning of a drill based on computed trajectories in a cadaveric scull.

Our results demonstrate that planning of minimally invasive multiport paths, providing access to the lateral skull base, is feasible. The presented planning and simulation of the trajectories are the first, necessary steps to successful minimally invasive multiport surgery of the otobasis. The translation from planning to actual cadaveric model testing is currently underway in our laboratory (Figure 11).

## 5. Conclusion

Our study shows that planning of minimally invasive multiport paths, providing access to the lateral skull base, is feasible. A future approach based on this planning may minimize iatrogenic damage and deliver a reproducible result with high outcome predictability, thereby improving patient satisfaction and safety. Cadaveric and clinical studies are required to further verify this approach to the lateral skull base.

## Figures and Tables

**Table 1:** The cumulative angle (CA) for each target point. The values represent the CA in degrees (internal auditory canal (IAC), round window (RW), and petrous apex (PA)).

Region	IAC	RW	PA
Median	98	112	40
STD deviation	27	30	12
Minimal value	13	42	19
Maximal value	126	153	62
